# Biosensor for human IgE detection using shear-mode FBAR devices

**DOI:** 10.1186/s11671-015-0736-3

**Published:** 2015-02-18

**Authors:** Ying-Chung Chen, Wei-Che Shih, Wei-Tsai Chang, Chun-Hung Yang, Kuo-Sheng Kao, Chien-Chuan Cheng

**Affiliations:** Department of Electrical Engineering, National Sun Yat-Sen University, Kaohsiung, 80424 Taiwan; Department of Computer and Communication, Shu-Te University, Kaohsiung, 82445 Taiwan; Department of Electronic Engineering, De Lin Institute of Technology, Taipei, 23654 Taiwan

**Keywords:** AlN, Shear mode, FBAR, Biosensor

## Abstract

Film bulk acoustic resonators (FBARs) have been evaluated for use as biosensors because of their high sensitivity and small size. This study fabricated a novel human IgE biosensor using shear-mode FBAR devices with *c*-axis 23°-tilted AlN thin films. Off-axis radio frequency (RF) magnetron sputtering method was used for deposition of *c*-axis 23°-tilted AlN thin films. The deposition parameters were adopted as working pressure of 5 mTorr, substrate temperature of 300°C, sputtering power of 250 W, and 50 mm distance between off-axis and on-axis. The characteristics of the AlN thin films were investigated by X-ray diffraction and scanning electron microscopy. The frequency response was measured with an HP8720 network analyzer with a CASCADE probe station. The X-ray diffraction revealed (002) preferred wurtzite structure, and the cross-sectional image showed columnar structure with 23°-tilted AlN thin films. In the biosensor, an Au/Cr layer in the FBAR backside cavity was used as the detection layer and the Au surface was modified using self-assembly monolayers (SAMs) method. Then, the antigen and antibody were coated on biosensor through their high specificity property. Finally, the shear-mode FBAR device with *k*_*t*_^2^ of 3.18% was obtained, and the average sensitivity for human IgE detection of about 1.425 × 10^5^ cm^2^/g was achieved.

## Background

In recent years, piezoelectric materials have been used in surface acoustic wave (SAW) resonators [1-5] and film bulk acoustic wave resonators (FBARs) [6-10] because of their low cost, low weight, and good reproducibility. However, the SAW resonator has high insertion loss and poor power handling capability. Hence, this study evaluated the potential applications of FBARs for biosensors because of their advantages, including low insertion loss, good power handling, and small size. The FBAR devices were constructed by a piezoelectric layer sandwiched between two electrodes and attached to substrate with backside cavity. Piezoelectric materials such as zinc oxide (ZnO) and aluminum nitride (AlN) have been used in FBAR devices for various applications [11-13] owing to their high acoustic velocity, better quality factor, and high electromechanical coupling coefficient. Besides, the piezoelectric materials of ZnO and AlN can be combined with silicon technologies in semiconductor fabrication processes [14,15]. Moreover, the acoustic velocity of AlN is 10,400 m/s, and it suits application for FBAR devices.

The acoustic wave of a FBAR has two transmittance modes: longitudinal mode and shear mode. In shear mode, acoustic wave energy does not dissipate in a liquid environment [16]. The backside cavity of FBAR can be used as the detection area for adsorbent matter. Under a mass loading, a frequency shift would be resulted in the frequency response of a FBAR [17]. The analysis methods were used for biosensor in liquid and tiny mass detection in air through the shear mode and longitudinal mode, respectively. Thus, FBAR devices were fabricated and constructed to evaluate their potential use in biosensors.

**Figure 1:**
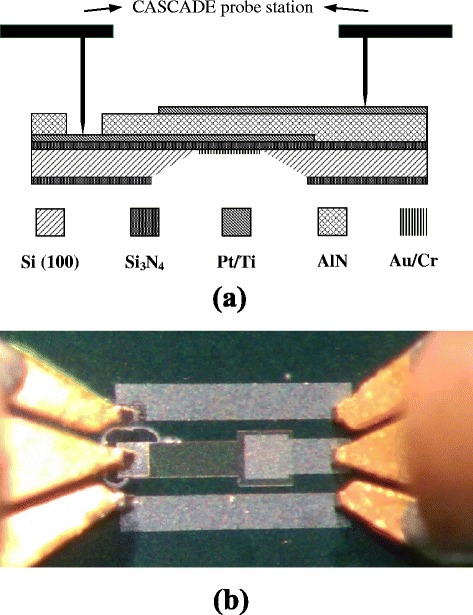
**The apparatus for evaluating the FBAR-based sensor devices. (a)** Schematic cross section view. **(b)** Front view.

According to the medicine journal report, it is estimated that as many as 1.4 billion people of allergy [18]. Hence, the marketable merit of anti-allergic agent is calculated to be 20 billion USD dollars [19]. The conventional detecting allergy methods focus on testing the concentration of immunoglobulins E (IgE) in human serum. The IgE in human immune system was used to resist exterior germs and virus, but overreactions of the human immune system can cause allergies. Furthermore, the traditional detecting allergy methods have some disadvantages such as time-consuming detecting process and large size and expensive detecting instrument [20]. Therefore, this investigation focuses on micro allergic sensor devices, due to the advantages, such as small size, low-cost, fast detecting process, etc. Besides, the apparatus for evaluating the FBAR-based sensor devices is shown in Figure 1a,b.

## Methods

### Fabrication of FBAR devices

**Figure 2:**
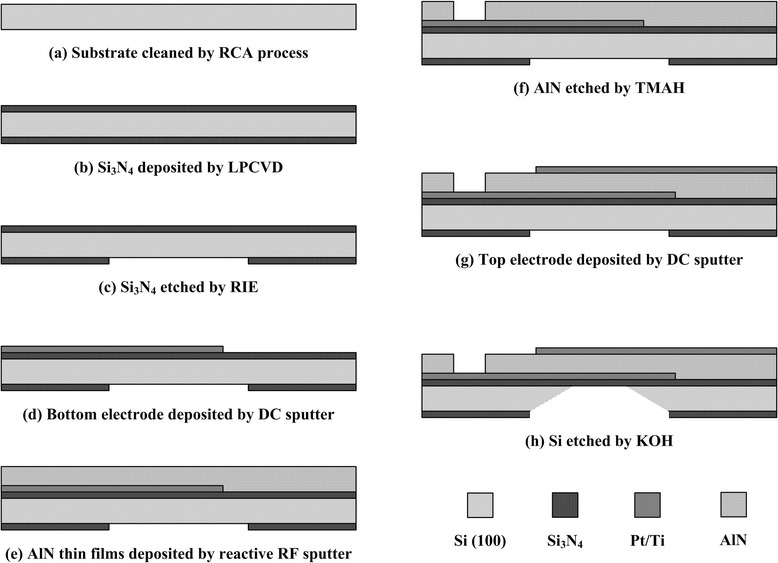
**The fabrication steps of FBAR devices.**

In this study, the FBAR devices for biosensors application were fabricated. Figure 2 showed the processes used to fabricate the shear-mode FBAR devices. The silicon nitride (Si_3_N_4_) was deposited on both sides of Si wafer by low-pressure chemical vapor deposition (LPCVD) as the supporting layer for the FBAR devices. The bottom electrodes, piezoelectric thin films, and top electrodes sandwiched structure is patterned by the photolithography process using four masking processes. The titanium (Ti) and platinum (Pt) layers were deposited on Si_3_N_4_/Si structure as bottom electrodes by a dual-gun DC sputtering system using 99.995% pure targets combined with first mask and lift-off method. The distance between target and substrate was fixed at 50 mm. As the base pressure was pumped down to 1 × 10^−6^ Torr, the film growth was carried out with working pressure of 3 and 1 mTorr for Ti and Pt, respectively. Then the high-quality AlN piezoelectric thin films were deposited on Pt/Ti layer using reactive radio frequency (RF) magnetron sputtering with off-axis deposition method. The Al target was 99.9995% pure, and the distance between target and substrate was fixed at 50 mm. As the base pressure was pumped down to 5 × 10^−7^ Torr, the sputtering conditions were set as working pressure of 5 mTorr, substrate temperature of 300°C, sputtering power of 250 W, and an off-axis to on-axis distance of 50 mm. To expose the bottom electrodes for electrical contact, AlN was wet-etched with 2.38% tetramethylammonium hydroxide (TMAH) using a second mask at room temperature. The top electrode can be obtained by the third patterning process after Pt/Ti was deposited on the AlN thin films. Finally, the backside of the structure was etched by combining the fourth mask and a 30% KOH solution to form the detection area. Therefore, the fabrication of the FBAR devices was then completed.

### Characteristics measurement

The characteristics of AlN thin films, including crystalline properties, preferred orientation, and cross-sectional morphologies were examined. The crystalline properties and preferred orientation of the AlN thin films were determined by X-ray diffraction scanning between 20° and 60° using a Siemens D5000 (Munich, Germany) with CuKα radiation. The surface morphologies and cross sections of AlN thin films were observed by field-emission scanning electron microscope (FESEM, JEOL-6700; JEOL Ltd., Akishima-shi, Japan). Finally, the frequency responses of FBAR devices with the biosensors were measured by HP8720 network analyzer.

### FBAR devices for biosensor applications

**Figure 3:**
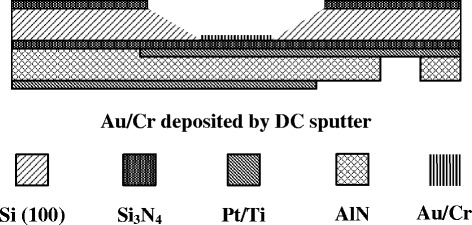
**The schematic diagram of a biosensor.**

For biosensor applications of the FBAR, Au/Cr thin films were deposited in the backside cavity of FBAR devices as the detection layer using a dual-gun DC sputtering system as shown in Figure 3. In the self-assembly monolayers (SAMs) method, the Au surface was modified by adsorption of thiolate (CH_3_(CH_2_)_n_SH).

The SAMs method was performed as follows:

- Step (1): Use oxygen plasma process for Au surface cleaning.
- Step (2): Inject cysteine solution (R.T., 1 h).
- Step (3): Inject deionized (DI) water and dry using N_2_ gas.
- Step (4): Inject glutaraldehyde solution (2.5%, R. T., 1 h).
- Step (5): Inject DI water and dry with N_2_ gas.

Then, the surface modification of FBAR devices were accomplished by the SMAs method. In the biosensors, human IgE was detected by using a coating process to detect antibody with antigen because of the high specificity between antigen and antibody. Hence, the coating process was performed as described in the literatures as follows [21-24]:

- Step (1): Wash with 200 μl phosphate-buffered saline (PBS) solution three times.
- Step (2): Dip 200 μl diluted mouse anti-human IgE antibody (37°C, 2 h).
- Step (3): Inject 200 μl, Tween-20 wash buffer three times.
- Step (4): Inject 200 μl, 10 wt.% bovine serum albumin (BSA) solution (37°C, 0.5 h).
- Step (5): Inject 200 μl, Tween-20 wash buffer three times.
- Step (6): Inject 200 μl, diluted human IgE antigen with 0.707 μg/ml concentration.

**Figure 4:**
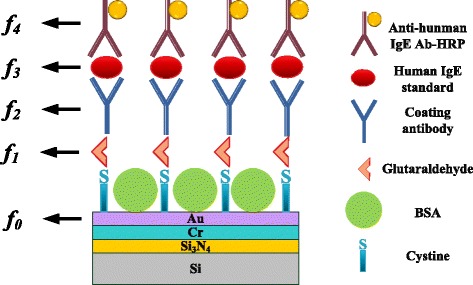
**The schematic diagram for the integration of cystamine SAM, glutaraldehyde, IgE antibody and antigen multilayer.**

In the backend process of step (2) to step (6), the sample were cleaned using DI water to remove excess liquid and then dried with N_2_ gas. Figure 4 schematically depicts the IgE antigen/IgE antibody/glutaraldehyde/the integrated cystamine SAMs multilayer [20].

After the above SAMs and coating processes, the frequency response was measured before and after anti-human IgE antibody linked with the human IgE antigen. Finally, the sensitivity (*S*_*m*_) of the biosensor was calculated using the following equation:

$$ {S}_m=\underset{\delta m\to 0}{ \lim}\left(\frac{\delta f}{f}\right)\left(\frac{1}{\delta m}\right), $$

where *δm* is the loading mass (9.1875 ng/cm^2^) and *δf* is the variation of the resonate frequency. Finally, the sensitivities of FBAR devices for human IgE detection were investigated.

## Results and discussion

### Structural and morphological properties of AlN thin films

**Figure 5:**
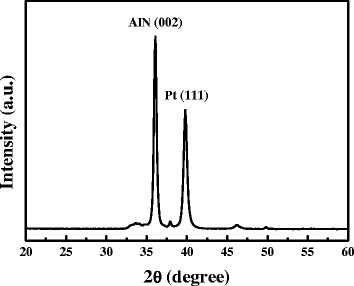
**The** ***θ*** **-2** ***θ*** **X-ray scans of the AlN thin film.**

**Figure 6:**
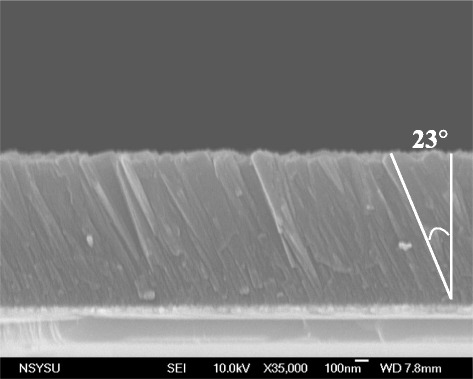
**The cross-sectional image of the AlN thin film.**

A highly *c*-axis orientation is the ideal piezoelectric property of a FBAR device. According to the literature, a 34.5° *c*-axis tilted piezoelectric thin film in FBAR device exits strongly shear-mode transmittance [25]. The optimized sputtering conditions for 23° *c*-axis tilted highly textured AlN thin films were obtained in our previous report [26], those were working pressure of 5 mTorr, substrate temperature of 300°C, sputtering power of 250 W, and the off-axis of 50 mm. Figure 5 shows the *c*-axis preferred orientation of AlN thin films with small full width at half maximum (FWHM). Besides, Figure 6 shows the cross-sectional images, which reveal columnar with 23°-tilted AlN thin films.

### Frequency responses of shear-mode FBAR devices

**Figure 7:**
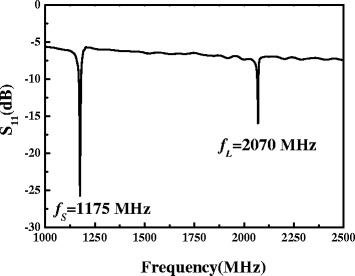
**The frequency response of a FBAR device without Au/Cr coatings.**

Figure 7 shows the frequency responses of the FBAR devices with 23°-tilted AlN thin films, in which the longitudinal mode and shear-mode exist at 2.07 (*f*_L_) and 1.175 GHz (*f*_S_), respectively. The ratio of *f*_L_ to *f*_S_ can be determined from the following relationship:

$$ \frac{f_{\mathrm{L}}}{f_{\mathrm{S}}}=\frac{V_{\mathrm{L}}}{V_{\mathrm{S}}}=\frac{\sqrt{\frac{C_{33}}{\rho }}}{\sqrt{\frac{C_{44}}{\rho }}}=\sqrt{\frac{C_{33}}{C_{44}}}=\sqrt{\frac{395\kern0.48em \mathrm{G}\mathrm{p}\mathrm{a}}{118\kern0.48em \mathrm{G}\mathrm{p}\mathrm{a}}}=1.83, $$

where *V*_L_ and *V*_S_ are the acoustic velocity, *C*_33_ and *C*_44_ are an elastic constant, and *ρ* is density of the wurtzite AlN. In this study, the practical acoustic velocity of longitudinal mode is 1.76 times than that of the shear mode, which is still consistent with the literature [25,27]. The electromechanical coupling coefficient (*k*_*t*_^2^) of shear mode is a numerical measurement of the conversion efficiency between electrical and acoustic energy in piezoelectric materials. The *k*_*t*_^*2*^ of the shear mode of the FBAR was calculated to be about 3.18%.

### Frequency responses of biosensors for human IgE detection

The Au/Cr thin films were adopted as detection layer using a dual-gun DC sputtering system, the oxygen plasma process was used to clean the surface of the Au layer in order to improve the hydrophilic properties of the contact area between the bio-drop and Au layer [28-32].

**Figure 8:**
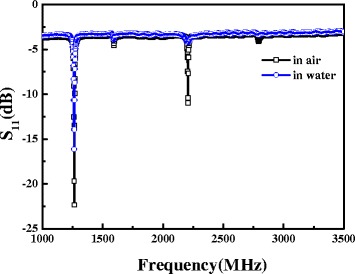
**The frequency response of a FBAR device in air and liquid environment.**

Besides, the analysis methods were used for biosensor in liquid and tiny mass detection in air through the shear mode and longitudinal mode, respectively. Figure 8 shows the frequency response of FBAR device in air and liquid environment. The longitudinal mode almost disappeared in liquid environment because of the decrease of quality factor (*Q*) which reduces the mass resolution substantially, whereas the shear mode maintains high readability. However, the literatures mentioned that the large reflection coefficient of longitudinal mode in solid and liquid interface which is the key factor result in the acoustic wave vanished. Therefore, the shear mode propagating in solid medium maintains its movement through a liquid environment [33-35]. The experimental and analytical results indicate that the longitudinal mode is the key indicator to identify the sensing environment, and the shear mode can be exploited in biosensor applications. Hence, FBAR devices with 23°-tilted AlN thin films are suitable for human IgE detection.

**Figure 9:**
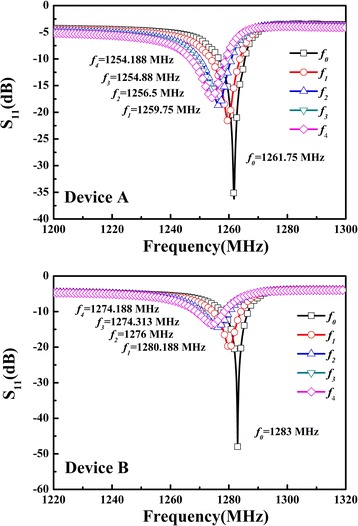
**The frequency responses of biosensors for human IgE detection, Device A and Device B.**

In this study, two devices of biosensors for human IgE detection were fabricated, and the frequency responses are shown in Figure 9. In Figure 9, *f*_0_, *f*_1_, *f*_2_, *f*_3_, and *f*_4_ are the resonate frequencies of the shear-mode FBAR devices without loading, treated with the SAMs method, combined with the anti-human IgE antibody, linked with the human IgE antigen, and terminated with the anti-human IgE HRP, respectively. The properties of shear-mode FBAR device adopted for the coating mass detection are demonstrated in Figure 9. In some literatures, the resonant frequency were also used to confirm the coating mass adhered on FBAR devices [20,36].

**Figure 10:**
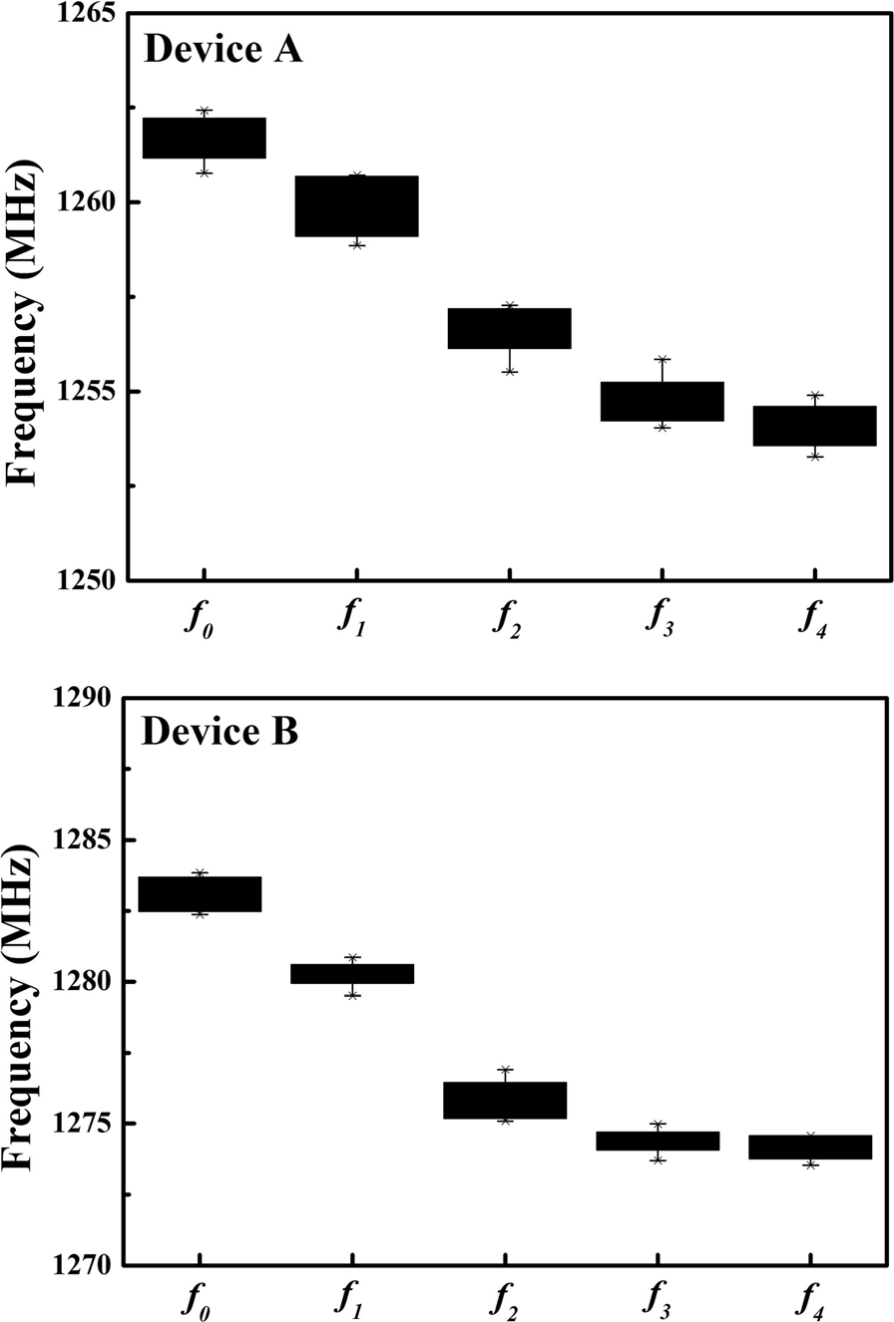
**The variations of frequency response step by step from** ***f***_**0**_ **to** ***f***_**4**_ **, Device A and Device B.**

Besides, after repeated testing, the variation of frequency response of the same device exhibited a tiny error of ±0.01%. In the bio-processes, the resonate frequency decreased the range of about 10 MHz which results from the bio-processes effect as SAMs, IgE antibody, IgE antigen, and HRP are added to the biosensor area. Figure 10 shows the variations of resonate frequency step by step from *f*_0_ to *f*_4_. It is confirmed that the matters have mutual bonding when coated on biosensor. However, the standard IgE reagent exist possible error value of ±0.5% in environment according to the official test reports and the enzyme-linked immunosorbent assay (ELISA).

To calculate the sensitivity (*S*_*m*_) of the shear-mode FBAR devices for human IgE detection, the *S*_*m*_ is calculated by ${S}_m=\underset{\delta m\to 0}{ \lim}\left(\frac{\delta f}{f}\right)\left(\frac{1}{\delta m}\right)$. Table 1 shows the calculated sensitivities for human IgE detection of two biosensor devices. The average sensitivity calculated for the shear-mode FBAR devices for human IgE detection was about 1.425 × 10^5^ cm^2^/g.

**Table 1:** **The frequency shift and sensitivity of biosensors**

	**Device A**	**Device B**
Frequency shift, *δf* (MHz)	1.62	1.687
Sensitivity, *S*_*m*_ (cm^2^/g)	1.41 × 10^5^	1.44 × 10^5^

The results of this study demonstrate that the proposed shear-mode FBAR device is highly promising for use in human IgE detection because of its high sensitivity, small size, low-cost, and rapid reaction process than conventional quartz crystal micro-balance (QCM) [37-41].

## Conclusions

This study fabricated shear-mode FBAR devices for biosensor applications. The AlN thin films and Pt/Ti were adopted as the piezoelectric and electrode layers, respectively, in FBAR devices. The AlN thin films were fabricated at a working pressure of 5 mTorr, substrate temperature of 300°C, sputtering power of 250 W, and off-axis of 50 mm. The resulted AlN thin films exhibited a strong *c*-axis orientation and 23°-tilted. The obtained shear-mode FBAR devices had a frequency response of 1.175 GHz and a *k*_*t*_^2^ of about 3.18%. For biosensor applications, the Au/Cr thin films were deposited on backside cavity of FBAR as bio-detection layer. The SAMs method was used for surface modification of Au thin films. Human IgE was detected by using a coating process to detect antibody with antigen. The average sensitivity for the shear-mode FBAR devices for human IgE detection was about 1.425 × 10^5^ cm^2^/g.
